# Fly Ash Application as Supplementary Cementitious Material: A Review

**DOI:** 10.3390/ma15072664

**Published:** 2022-04-05

**Authors:** Guanlei Li, Chengke Zhou, Waqas Ahmad, Kseniia Iurevna Usanova, Maria Karelina, Abdeliazim Mustafa Mohamed, Rana Khallaf

**Affiliations:** 1 Luohe Vocational Technology College, Luohe 462300, China; dayanta100@sina.com; 2 Department of Civil Engineering, COMSATS University Islamabad, Abbottabad 22060, Pakistan; 3 Peter the Great St. Petersburg Polytechnic University, 195291 St. Petersburg, Russia; usanova_kyu@spbstu.ru; 4 Department of Machinery Parts and Theory of Mechanisms, Moscow Automobile and Road Construction University, 125319 Moscow, Russia; karelinamu@mail.ru; 5 Department of Civil Engineering, College of Engineering in Al-Kharj, Prince Sattam bin Abdulaziz University, Al-Kharj 11942, Saudi Arabia; a.bilal@psau.edu.sa; 6 Building & Construction Technology Department, Bayan College of Science and Technology, Khartoum 11115, Sudan; 7 Structural Engineering and Construction Management, Faculty of Engineering and Technology, Future University in Egypt, New Cairo 11845, Egypt; rana.khallaf@fue.edu.eg

**Keywords:** cementitious composites, fly ash, supplementary cementitious material, mechanical properties, durability, microstructure

## Abstract

This study aimed to expand the knowledge on the application of the most common industrial byproduct, i.e., fly ash, as a supplementary cementitious material. The characteristics of cement-based composites containing fly ash as supplementary cementitious material were discussed. This research evaluated the mechanical, durability, and microstructural properties of FA-based concrete. Additionally, the various factors affecting the aforementioned properties are discussed, as well as the limitations associated with the use of FA in concrete. The addition of fly ash as supplementary cementitious material has a favorable impact on the material characteristics along with the environmental benefits; however, there is an optimum level of its inclusion (up to 20%) beyond which FA has a deleterious influence on the composite’s performance. The evaluation of the literature identified potential solutions to the constraints and directed future research toward the application of FA in higher amounts. The delayed early strength development is one of the key downsides of FA use in cementitious composites. This can be overcome by chemical activation (alkali/sulphate) and the addition of nanomaterials, allowing for high-volume use of FA. By utilizing FA as an SCM, sustainable development may promote by lowering CO_2_ emissions, conserving natural resources, managing waste effectively, reducing environmental pollution, and low hydration heat.

## 1. Introduction

Industrial solid waste makes up a sizable portion of human-generated wastes, which come in a vast range of forms and are highly complex in nature [1,2,3]. Heavy metals are found in the majority of industrial wastes, such as red powder, metal cleaning, and radioactive waste [4,5,6]. Inappropriate handling of solid industrial waste can lead to leachate penetrating soil and groundwater, causing environmental irreversibility and endangering human health [7,8,9]; moreover, global warming and climate change are the two most serious environmental problems caused by CO_2_ emissions [10,11,12]. The construction industry substantially impacts the environment, accounting for a significant portion of CO_2_ emissions [13,14,15]. Each ton of cement produced emits about 0.8 tons of CO_2_ [16,17,18], and cement production is increasing globally [19] due to the increasing demand for concrete [20,21,22]. Researchers worldwide are constantly on the lookout for new materials that can be utilized in place of, or in addition to, cement [23]. Since the last decade, the application of supplementary cementitious materials (SCMs) such as silica fume, fly ash (FA), slags, etc., as a cement replacement has been emphasized [24,25,26]. SCMs hydrate cement hydraulically or pozzolanically in pore solution [27,28,29]. Thus, utilizing industrial solid waste in construction as SCMs is an effective approach for eco-friendly construction [30]; it could reduce cement demand, reduce CO_2_ emission, and solve waste management problems. From the various kinds of industrial byproducts that can be used as SCMs, the most common is FA.

FA is a byproduct of coal combustion that is accumulated at the top of boilers, particularly in coal-fired power plants [31,32]. The omitted mineral particles or mineral materials within the coal liquefy, evaporate, consolidate, or agglomerate during/after burning. By rapidly cooling in the post-combustion portion, sphere-shaped, amorphous FA grains are created due to surface tension force. When the entrapped volatile matter reaches a high temperature, it expands inside, forming a hollow cenosphere. Some residues may crystallize, while others may become glassy, reliant on the composition of residues and the heating/cooling circumstances [33]. FA is considered an SCM that is used in place of cement in cementitious materials [34,35]. FA increases workability, decreases the hydration heat, and thermal cracking in cementitious materials at initial ages, and improves the mechanical and durability characteristics of cementitious composites, mostly at later ages [36,37]. The application of FA is also being investigated in the manufacture of geopolymer concrete [38,39,40]; however, this study is limited to reviewing their utilization in cementitious composites.

The amorphous silica in FA reacts with the calcium hydroxide to form calcium-silicate-hydrate (CSH) [41]. FA’s pozzolanic reaction boosts its utility not only in concrete but also in a variety of other construction applications [42]. Due to the pozzolanic process, the strength gain lasts significantly longer than with normal concrete [43]. Additionally, FA increases the workability of concrete by reducing bleeding [44]. FA has been shown to improve the long-term compressive strength (CS) of normal and recycled aggregate concrete [45]. Microstructural examination of FA samples following early curing reveals an abundance of un-hydrated spherical FA particles. Despite this, after a year of curing, the microstructure of FA samples appeared to be very compact, with no evidence of dehydrated FA particles [46]. FA requires a longer period of time to hydrate. As a result, during the initial phases of curing, low CS has been found. The strength development of FA, on the other hand, is dependent on its chemical and physical characteristics. It has been observed that FA with a fine particle size distribution had a better CS than FA with a coarse particle size distribution [47]. The binder causes the concrete to shrink during the hydration process, and excessive shrinkage can result in severe cracks in the concrete structure. FA is beneficial for shrinkage mitigation [48]. It has been noted that the use of large volume FA in concrete, specifically 50% replacement of cement with FA, resulted in a 30% reduction in shrinkage when compared to ordinary concrete [49].

The use of FA in low (<30%) and high (>30%) volume in concrete is a pioneering move that has already altered the worldwide concrete industry’s approach. Mostly, FA is disposed of in landfills, which has had severe implications, the majority of its portion has been successfully utilized in the concrete industry for the last three to four decades. Additionally, the frequent generation of FA has compelled government officials and experts to develop a more dependable method of consuming it, while the application of FA to the development of sustainable concretes will almost certainly alter the future building industry [50,51,52]. Even though FA has been extensively investigated over the last few decades, experts have discovered some inconsistent results regarding the mechanical and durability characteristics of concrete. The chemical and physical features of FA have a major effect on the mechanical and durability characteristics of concrete. Additionally, the characteristics of FA vary depending on the source. This study focused on reviewing the mechanical, durability, and microstructural characteristics of FA-based concrete; the various factors influencing the aforesaid properties are highlighted, and limitations associated with the use of FA in concrete are described. Based on the review of the literature, possible solutions to the limitations are provided, and future research is directed to the application of FA in larger quantities.

## 2. Properties of Fly Ash

### 2.1. Physical and Chemical Properties

**Figure 1:**
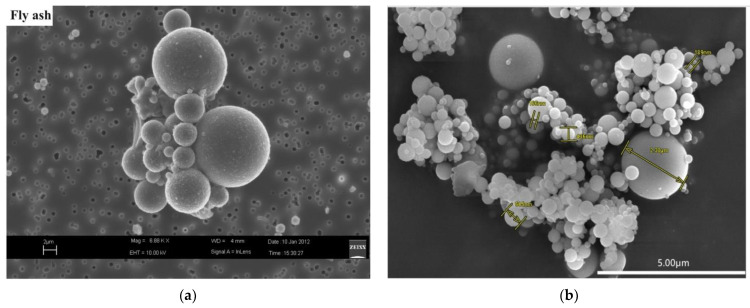
Micrograph of fly ash: (**a**) [70]; (**b**) [71].

FA is a primary solid waste generated by coal-fired energy plants, and these plants are looking for economically viable ways to dispose of it. FA particles are generally spherical, solid/hollow in nature, mainly glassy (amorphous), with particle sizes varying from <1 µm to 150 µm [53,54,55]. The scanning electron microscopy (SEM) micrographs of the FA are shown in Figure 1. FA has a specific gravity of 2.1 to 3.0 [56] and a specific surface area of 170 to 1000 m^2^/kg [57]. FA can range in color from tan to grey to black, based on the quantity of unburned carbon present [35,58]. Besides the environmental advantages of waste disposal and CO_2_ reduction [59,60], FA increases workability [61], decreases the hydration heat and thermal cracking in concrete at the initial stage [62], and improves the performance of cementitious materials, especially at later stages [36,63]. Regardless of the advantages of FA, 100% application of FA is not possible due to a variety of reported limitations [64]. The ASTM categorizes FA into two categories: “C” and “F” [65]. FA classified as “Class F” is mostly generated by burning anthracite or bituminous coal containing SiO_2_, Al_2_O_3_, and Fe_2_O_3_ concentrations greater than 70%. While “Class C” FA is generated by burning lignite or sub-bituminous coal that consists of 50% to 70% of the aforementioned chemicals [66]. Class F is a typical pozzolan and composed of silicate glass that has been modified with aluminum and iron [67]. CaO amount is less than 10% in “Class F” FA [68]; thus, to form CSH through pozzolanic reaction, Ca(OH)_2_ formed during cement hydration is required; therefore, the chemical composition of FA performs a major part in determining its performance in cementitious composites [69]. The range of element oxide concentrations found in “Class F” and “Class C” FA has been listed in Table 1. As can be seen, there is a considerable difference in the element oxides contained within a single kind of FA, which might be ascribed to differences in source, processing conditions, and so on. There is a crucial need to utilize more FA in the construction materials due to the increase in FA production globally.

### 2.2. Cementing Efficiency and Pozzolanic Properties

Smith [72] proposed the notion of cementing factor (k) in order to develop a reasonable approach for incorporating FA into cement/concrete. Cementing efficiency can be employed to ascertain the overall quality, durability, and performance of composites. In general, FA has a low cementing efficiency at initial stages and acts more such as filler, but the pozzolanic feature turns out to be efficient at later ages, causing a significant increase in strength [73,74,75]. This clearly indicates that the pozzolanic reaction improves the cementing efficiency of FA with age. According to Smith [72], “the FA mass might be considered similar to the cement mass in terms of CS development.” In other words, “k” is a factor that accounts for the variation among the contribution of cement to the development of a particular property and the contribution of mineral admixtures. CS tests are frequently used to measure this cementing efficiency due to their simplicity and repeatability. The “k” value of FA is determined by a variety of its intrinsic qualities, including physical properties such as particle size, distribution, and shape, as well as chemical composition [76]. Additionally, it was also reported that the “k” factor is dependent on other parameters such as the curing time, the concrete strength, and the FA type [77]; therefore, it was also discovered that the “k” value is dependent on external factors such as the water/cement ratio (*w*/*c*). They stated that for conventional FA, “k” is a function of the *w*/*c* and that the cementing efficiency of FA tends to decline as the *w*/*c* increases [78]. On the contrary, Smith [72] asserted that it is unaffected by the *w/c*.

Apart from cementing efficiency, pozzolanicity is another critical term in the context of FA concrete. Among the numerous favorable benefits of FA in cement/concrete, the pozzolanic effect is believed to be the most important [66]. The pozzolanic reaction is mostly dependent on the Al_2_O_3_ and SiO_2_ content of FA and is stimulated by the Portlandite generated during cement hydration to generate a more hydrated gel. This gel plugs the capillary pores in the matrix, increasing its strength [79]. As a result, FA’s reactivity is greatly dependent on its chemical properties; however, all pozzolanic materials are made of aluminosilicate glass that combines with Ca(OH)2 formed during hydration of cement to yield hydration products [80].

## 3. Properties of Composites Containing Fly Ash

### 3.1. Workability

FA has plasticizing properties that improve the workability of the composites [81]. Lee et al. [82] reported the subsequent factors as possible reasons for FA’s plasticizing effect. Firstly, increased composite volume due to FA’s lower density than cement. Secondly, FA decreases the flocculation of cement grains because of the dilution effect. Thirdly, the slower reaction rate of FA reduces hydration product growth at the initial time. Besides these causes, the spherical shape of FA grains facilitates the movement of nearby particles by the ball bearing effect, particularly at high replacement levels. Thus, FA can be a more cost-effective method with a low environmental effect to increase the workability than chemical superplasticizer [83]. Bentz et al. [84] also validated the positive impact of FA on the workability of the mix. The type of FA used also has a substantial impact on the workability of composites. According to Ponikiewski and Golaszewski [85], high calcium FA has a detrimental effect on workability, which adversely influences the mechanical strength of composites. The fresh state characteristics of a mix mostly depend on the flowability of cement paste, which is affected by a variety of aspects such as water-binder ratio (*w*/*b*), type, and quantity of SCM [86]. Conversely, some researchers reported a drop in the workability of mixes with FA addition at higher amounts [87]. The decrease in workability might be the high-water demand due to the smaller size and larger surface area of FA.

Lee et al. [82] highlighted the following aspects as possible explanations for FA’s plasticizing action. To begin, increased paste volume due to FA’s lower density than cement. Second, FA lowers the flocculation of cement particles due to the diluting effect. Thirdly, because of the FA’s slower reaction rate as a result of the lowered development of hydration products at an initial age. In addition, the spherical shape of FA grains facilitates the movement of nearby fragments via the ball bearing effect, particularly at high replacement levels; thus, FA can be a more cost-effective method of increasing flowability than chemical superplasticizers [83]. Bentz et al. [84] also validated the favorable effect of FA on the fluidity of the mix. As previously observed [82], replacing cement with FA reduces yield stress due to the decreased density of FA and hence decreases the number of flocculated cement grain to cement particle contacts. The FA type utilized has also a considerable effect on the fresh properties. According to a study, high calcium FA has a detrimental effect on workability, which in turn has a negative effect on strength and durability [85]. The fresh state performance of a concrete is primarily dependent on the flowability of the cement paste, which is influenced by a variety of elements such as *w*/*b*, type, and dose of SCM [88].

Apart from these parameters, prior research has also demonstrated that the packing density of the cement-based composites also has a significant role in determining the cement paste’s flowability, particularly at low *w*/*b* ratios [82,89]. Essentially, increased packing density results in decreased water demand, which results in increased water being released (after voids filling) to cover the solid fragments and lubricate the cement paste [90]; however, a higher specific surface area significantly increases the amount of solid surface area that can be covered with water [91,92,93]. These simultaneous actions of tiny fillers can enhance packing density while decreasing the quantity of surplus water per surface area; thus, to achieve a balance between the desired increase in packing density and the unwanted increase in surface area, a filler that is finer than cement but coarser than nano silica/silica fume is required [94]. This indicates that the fineness of the FA impacts the end material’s properties. As a result, it has been concluded that some studies show lower water required for concrete workability due to the refined pores and spherical morphology of FA; others report a higher water requirement due to its increased surface area. This well-documented incompatibility between water demand and FA usage must be rectified.

### 3.2. Compressive Strength

Numerous tests are used to determine the concrete performance, but CS is often regarded as the most critical. CS tests provide a clear indication of the varied properties of concrete. The literature established that CS is related to a variety of mechanical and durability attributes directly or indirectly [95]. In other words, CS and the quality of concrete are inextricably related. FA’s physical properties, particularly its size and shape, have a substantial effect on the performance of cement-based materials. Additionally, the chemical composition has been considered a base to ascertain the appropriateness of FA for use as SCM [96]. Thus, the hydration process of the FA-cement mix is strongly affected by the intrinsic characteristics of FA, for instance, crystalline structure, chemical and physical properties [97], as well as external factors such as *w*/*b*, replacement ratio, and curing temperature. FA fineness is a major factor [98] in controlling the appropriateness of FA in cementitious composites, as the FA grain size has a substantial impact on the performance of composites [99]. The packing and nucleation effects on the cement hydration are highly reliant on the particle size of the FA used [100]. Chindaprasirt et al. [100,101,102] conducted a thorough investigation to examine the effect of the fineness of FA on the composites’ properties; they reported that using finer FA resulted in an increase in CS. It was discovered that coarse FA is less reactive and needs extra water, producing a more porous mortar. The detrimental effects of coarser FA are described as a cause of decreased strength. Numerous findings have indicated that the application of FA impairs the early-age strength development of composites [82,103,104,105]; however, FA generally enhances the strength and durability of composites over time, as it consumes the Ca(OH)_2_ produced during the hydration of cement and makes secondary hydrates, for example, CSH [106].

**Figure 2:**
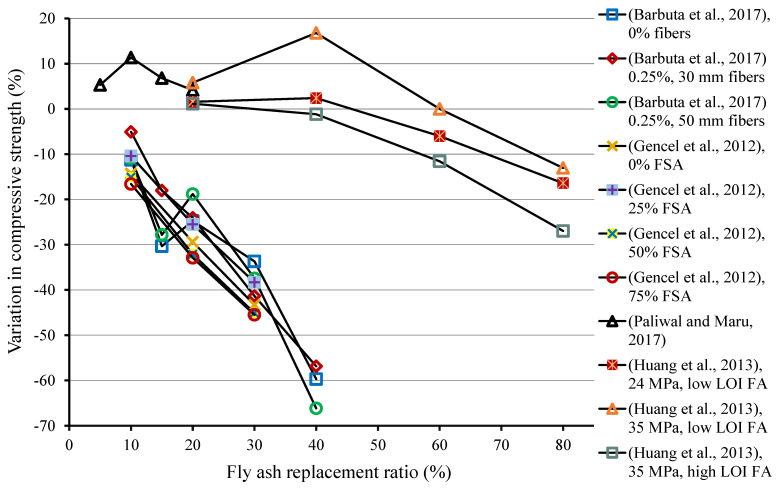
Influence of fly ash as cement replacement on 28-days compressive strength of composites. FSA: ferrochromium slag aggregate [107,108,109,115].

The quantity of FA used as cement replacement in composites also affects their properties. The 28-days CS results of specimens at various replacement levels of FA have been provided in Table 2. In addition, the influence of FA replacement ratio, based on past studies, on 28-days CS of composites compared to the reference samples without FA has been shown in Figure 2. Barbuta et al. [107] observed a decrease in CS with the use of FA, and a higher quantity of FA as cement replacement resulted in greater loss of CS. The samples without fibers showed a decrease in CS by 11.3%, 30.4%, 24.8%, 33.7%, and 59.7%, with FA content of 10%, 15%, 20%, 30%, and 40%, respectively, related to the controlled sample. A comparable pattern was also noticed with the samples containing fibers. Gencel et al. [108] assessed the impact of FA on the properties of composites using 10%, 20%, and 30% FA in place of cement. A reduction in CS was observed with the use of FA, compared to the reference mix, also shown in the figure. The reduction in CS was more at higher FA contents. Huang et al. [109] studied the impact of two kinds of FA depending on loss on ignition (LOI) amount, i.e., low LOI (4.6%) and high LOI (7.8%) FA. The outcomes discovered that utilizing low LOI FA at lower proportions enhanced the CS. The maximum increase in CS was examined at 40% content of FA having 16.8% higher CS than that of the reference sample; however, at increased proportions of FA, the CS reduced, which may be ascribed to the finer grain size in low LOI FA, which made the microstructure denser and more compact. The CS reduced with the addition of high LOI FA was because of greater particle size and lower pozzolanic activity; it was also reported that the CS of composites containing higher contents of FA was improved at a later age (1 year) compared to the controlled sample because of the slow pozzolanic reaction.

The effect of FA addition has been investigated in self-compacting concrete (SCC). For example, substituting 35% FA for cement results in a 10% reduction, but substituting 55% FA leads to a 24% reduction compared to the control SCC mix [110]. Similarly, a reduction of approximately 46% and 35% have been seen in containing 50% FA mix, after 7 and 90 days of curing, respectively, when compared to a control SCC mix; moreover, at a 70% FA incorporation level in SCC, a severe reduction of approximately 63% and 47% was seen after 7 and 90 days of curing, respectively [111,112]. The presence of cement additives has a considerable effect on the CS of FA-based SCC. The addition of cement additives improves the performance of FA-based SCC mixtures at both low and high curing temperatures. Silica fume, metakaolin, and limestone filler have all been used previously to increase the CS of SCC mixes. At 90 days, a reduction of nearly 29%, 42%, and 15% was seen for a 50% level of FA with limestone filler (15%), metakaolin (20%), and silica fume (10%) in SCC, respectively [111,113,114].

**Table 2:** Compressive strength (28-days) of composites containing fly ash.

Reference	Replacement Ratio (%)	Compressive Strength (MPa)
Barbuta et al. [107], without fibers	0	33.4
	10	29.7
	15	23.3
	20	25.2
	30	22.2
	40	13.5
Barbuta et al. [107], with 0.25% and 30 mm long fibers	0	33.4
	10	31.8
	15	27.4
	20	25.4
	30	19.6
	40	14.4
Barbuta et al. [107], with 0.25% and 50 mm long fibers	0	33.4
	10	29.8
	15	24.1
	20	27.2
	30	20.9
	40	11.3
Gencel et al. [108], with 0% FSA	0	52.2
	10	44.7
	20	36.8
	30	29.6
Gencel et al. [108], with 25% FSA	0	50.7
	10	45.4
	20	37.8
	30	31.3
Gencel et al. [108], with 50% FSA	0	53.9
	10	45.8
	20	36.6
	30	29.6
Gencel et al. [108], with 75% FSA	0	55.6
	10	46.4
	20	37.3
	30	30.3
Paliwal and Maru [115]	0	26.4
	5	27.8
	10	29.4
	15	28.2
	20	27.5
Huang et al. [109], 24 MPa concrete and low LOI fly ash	0	25.0
	20	25.4
	40	25.6
	60	23.5
	80	20.9
Huang et al. [109], 35 MPa concrete and low LOI fly ash	0	34.5
	20	36.5
	40	40.3
	60	34.5
	80	30.0
Huang et al. [109], 35 MPa concrete and high LOI fly ash	0	34.5
	20	34.9
	40	34.1
	60	30.5
	80	25.2

FSA: ferrochromium slag aggregate, LOI: loss on ignition.

### 3.3. Split-Tensile Strength

**Figure 3:**
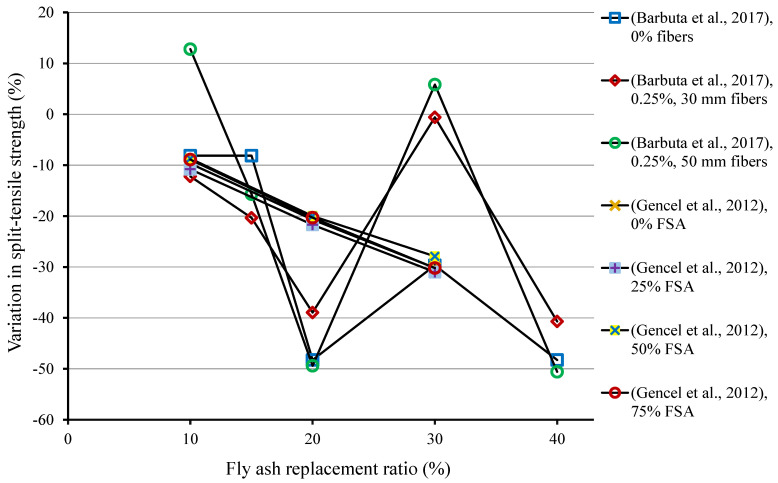
Influence of fly ash as cement replacement on 28-days split-tensile strength of composites. FSA: ferrochromium slag aggregate [107,108].

Another essential mechanical characteristic of concrete is its tensile strength, which has a significant effect on the extent and size of cracking in concrete structures. Because concrete is weak in tension, it is critical to do a pre-evaluation of their split-tensile strength (STS) [116,117]. The use of FA in cementitious composites has a detrimental effect on STS. The 28-days STS results of composites containing FA as SCM are displayed in Table 3. Figure 3 is generated on the data acquired from the literature depicting the variation in 28-days STS due to the replacement of cement by FA. Mostly, a reduction in STS is observed, especially at higher replacement ratios. From the experimental data performed by Barbuta et al. [107], the samples without fibers showed a decrease in STS by 8.1%, 8.1%, 48.3%, 29.6%, and 48.3% with FA content of 10%, 15%, 20%, 30%, and 40%, respectively, as compared to the control sample. The sample containing fibers (0.25% and 50 mm long) and 10% FA showed 12.8% higher STS when compared to the control sample; however, with the further addition of FA, STS was reduced. Gencel et al. [108] studied the combined effect of FA as SCM and ferrochromium slag as an aggregate replacement on STS of composites. They also reported decreasing trend with the addition of FA. The STS of specimens without ferrochromium slag was reduced by 9.7%, 20.7%, and 30.2%, with FA content of 10%, 20%, and 30%, respectively, compared to the sample without FA. A similar pattern of decreasing STS with FA addition was also noted in specimens containing ferrochromium slag as aggregate replacement.

STS was increased at higher curing ages in comparison to lower curing ages, as examined in most previous findings. According to published reports, FA interacts with calcium ions from Ca(OH)_2_ to CSH, the binder phase. Due to the lack of CSH and Ca(OH)_2_ in FA-containing concrete, it is unable to build early age strength [118,119]; moreover, the addition of FA reduces the STS of SCC mixtures due to its intrinsic propensity to reduce water [112,120]; however, in FA-based mixes, a considerable increase in STS has been seen with an increase in curing time, despite the presence of minor decrements. Due to the extensive research conducted to date on the effect of curing on FA-based mixes, similar improvements have been noticed in various studies [121].

### 3.4. Flexural Strength

**Figure 4:**
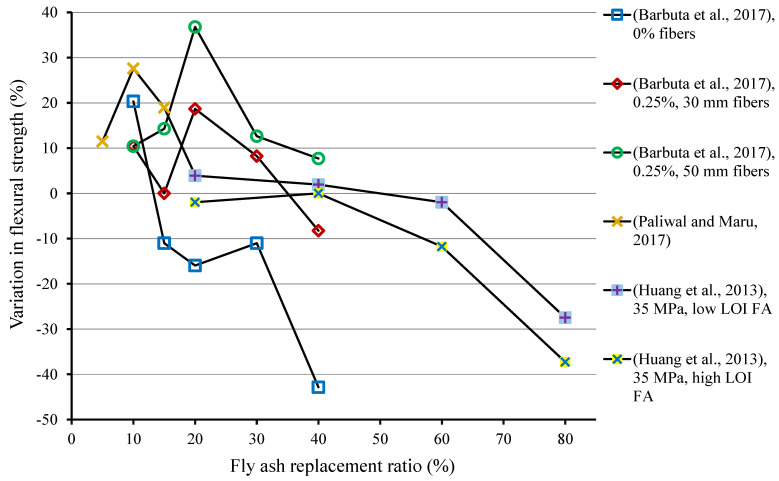
Influence of fly ash as cement replacement on 28-days flexural strength of composites [107,109,115].

The review of the past studies revealed that using FA at lower replacement levels can improve the flexural strength (FS) of composites, as shown in Table 4. Figure 4 is generated, indicating the percentage variation in 28-days FS of specimens at various replacement levels of FA. The improvement of 20.33% was observed in the FS when 10% FA was used SCM, while further increase in FA content decreased the FS compared with the reference specimen [107]. The results of Barbuta et al. [107] of specimens with 30 mm long fibers exhibited improvement in FS of 10.4%, 0%, 18.7%, and 8.2% with FA content of 10%, 15%, 20%, and 30%, respectively. While FS reduced by 8.2% at 40% replacement of FA. The specimens containing 50 mm long fibers showed 10.4%, 14.3%, 36.8%, 12.6%, and 7.7% increase in FS when 10%, 15%, 20%, 30%, and 40% cement was replaced by FA, respectively. Hence, it resulted that using a higher amount of FA has a negative influence on FS [107,108], as shown in Figure 4. Paliwal and Maru [115] noted maximum FS at 10% FA content as cement replacement. It can be concluded that the size, type, chemical composition, and content of FA used in cementitious composites have distinct effects on their mechanical properties. The finer particle size improves, while coarser particle size reduces the strength of composites [109]. Additionally, lower content of FA improves while higher FA content reduces the strength of composites [107,108,109]; hence, finer FA and a lower replacement ratio are preferable.

### 3.5. Durability

#### 3.5.1. Chloride Penetration

**Figure 5:**
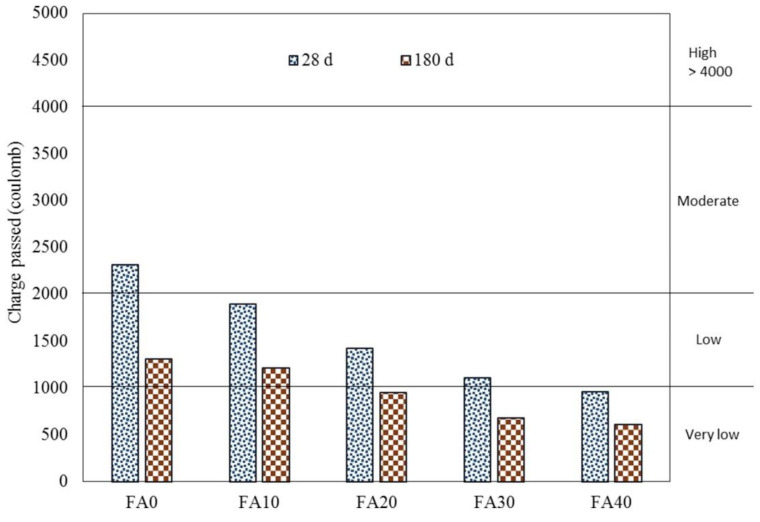
Chloride penetration of fly ash based-concrete at 28 and 180 days of curing [122].

The addition of FA also enhances the durability performance of cementitious composites. Saha [122] investigated the durability properties of concrete containing FA at various replacement levels and curing ages of 28 and 90 days. The results of the chloride ion penetration test revealed a decrease in penetration depth with FA addition (see Figure 5). At 28-days of curing, the chloride ion penetration depth reduced by around 18%, 39%, 52%, and 61% at FA content of 10%, 20%, 30%, and 40%, respectively. After 180 days, chloride penetration decreased marginally for all samples. The incorporation of 10%, 20%, 30%, and 40% FA content resulted in chloride ion penetration reduction of about 7%, 27%, 48%, and 53%, respectively, compared to the control mix. While the volume of the paste remains constant for mixes, the penetration of chloride ions into the matrix is determined by two fundamental factors, including the interlinking pores of the matrix and the free hydroxyl ion in the pore solution. Due to the finer particle size of FA, it may have minimized the interconnecting spaces and decreased the chloride ion penetration. FA can help concrete perform better over time in terms of CS, STS, FS, porosity, chloride penetration, creep, capillary absorption, drying shrinkage, surface scaling, and sulphate attack. Mainly, FA enhanced CS marginally but greatly increased the long-term STS and FS of concretes [123,124,125]. Class F FA in concrete provided more CS and chloride penetration resistance than Class C FA, and the maximum long-term CS was achieved for a FA concrete (67% Class F FA) at the age of seven years, along with exceptional surface scaling resistance [126]. Even when exposed to a sea environment for five years, FA concrete demonstrated strength development. Additionally, utilizing FA in concrete can help prevent chloride permeability and rusting of embedded steel bars [127]. All these long-term advantages can be ascribed to the pozzolanic nature of FA, which improves the amount of CSH, causing cross-linking hydrates at the molecular level and a compact and crack-free microstructure, thereby enhancing durability [125].

#### 3.5.2. Shrinkage

One of the main causes of concrete cracking is the strains caused by shrinkage. While the stresses created by restricted shrinkage have no effect on the structure’s integrity, they do raise the likelihood of durability issues [128,129]. While drying shrinkage occurs as a result of the concrete losing water, autogenous shrinkage occurs as a result of a variation in macroscopic volume when no moisture is transported to the adjacent environment. As a result, composites’ shrinkage must be considered cumulative, taking into account both drying and autogenous deformations. According to reports, volume variation because of shrinkage can frequently be addressed utilizing fillers such as FA [130]. A previous study found that SCM-based composites displayed more drying shrinkage than conventional cement-based composites [131]. Particularly, mixes comprising FA shrink more during the drying process than mixtures, including micro silica and slag cement. The pore structure of a concrete mixture containing SCMs such as FA, micro silica, and slag cement is more refined than that of a concrete mixture comprising only cement. As a result, these mixtures have a greater number of smaller capillary spaces, and thus, the water removal from these pores might result in increased drying shrinkage [94]. Another study also noted that composites with a greater proportion of SCMs have a finer pore structure, which may enhance free shrinkage proportionately [132]. Specifically, the loss caused by autogenous shrinkage can be considerably decreased by the inclusion of FA in composites [133]. Both Class C and Class F FA are thought to be beneficial for minimizing drying shrinkage [134]. The inclusion of FA reduces shrinkage by densifying the mix and preventing internal moisture evaporation [135]. Another reason for the limited shrinkage documented in the literature is the existence of un-hydrated FA grains in the matrix, which act as fine aggregates [136]; however, a few studies have found that FA with a smaller particle size than cement increases autogenous shrinkage [137]. A small-sized FA reduces the space between particles, which reduces the pore size in the paste and, as a result, capillary pressure increases in the paste while consuming water in the hydration process. While some research finds decreased shrinkage as a result of FA addition, a few others report an increase in shrinkage properties as a result of FA incorporation; thus, the influence of varying quantities of distinct FA types on the shrinkage of cement-based materials must be investigated to identify how shrinkage is reduced in FA concrete.

#### 3.5.3. Sulfate Resistance

The effect of FA on the resistance of mortar and concrete to sulphate attack has been widely studied. The significant range in performance of FA cement blends is due to the variety of FA kinds and compositions, as well as changes in mix proportions and construction techniques. In general, low Ca FA is more resistant to sulphate than high Ca FA because it can consume more Ca(OH)_2_ from the hydrated cement paste, generating more sulfate-resistant CSH without incorporating additional reactive phases present in high Ca FA that can accelerate sulfate-induced deterioration, while high Ca FA can hydrate independently during the generation of additional Ca(OH)_2_, hence accelerating sulfate-induced deterioration [138]. Apart from changes in calcium concentration, the amount of oxides in FA, including silica, alumina, and iron, as well as their amorphous and crystalline forms, has been demonstrated to have a substantial effect on their sulphate attack efficacy. FA containing less than 5% CaO is anticipated to have no reactive alumina and hence would not react with external sulphates to create expansive ettringite crystals [139]. Most of the studies reported an increase in sulphate resistance of the concrete with FA addition [140,141,142].

#### 3.5.4. Water Absorption

**Figure 6:**
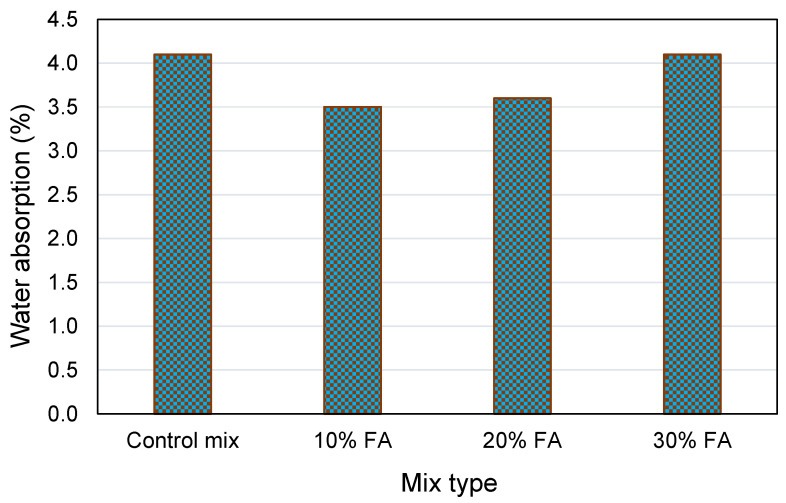
Water absorption of concrete at various contents of fly ash [145].

Water absorption (WA) is a feature of cementitious materials that are directly associated with its durability or long-term behavior. The existence of pores, cracks, and fissures in the matrix increases WA, which influences the mechanical and other durability aspects. In general, an increase in WA associated with an increase in FA indicates an increase in the volume of accessible pores [114,143]. Pitroda et al. [144] concluded that when 10% FA is replaced with cement, the WA of concrete decreases. Additionally, they discovered an increasing trend in WA as the level of cement replaced by FA increased by more than 10%. The WA of FA-incorporated concrete, on the other hand, was found to be greater than those of conventional concrete. In contrast, Hatungimana et al. [145] noticed a reduction in the WA of concrete with FA addition, as depicted in Figure 6. They reported that WA values increased as the amount of FA substitution increased, probably because the 28-day curing period was insufficient to complete the pozzolanic reaction; however, at 10% and 20% FA content, the WA of the samples was reduced by 14.6% and 12.2%, respectively, compared to the control mix. Whereas at 30% FA content, the FA concrete sample exhibited a comparable WA capacity to that of the control mix. Finally, the results indicated that FA could be employed as an SCM with some prudent engineering judgments [144].

### 3.6. Microstructure

**Figure 7:**
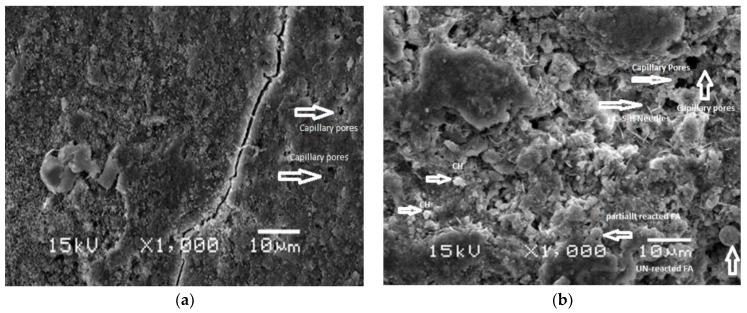
Microstructure of samples: (**a**) Without fly ash; (**b**) With 30% fly ash [147].

Gunasekara et al. [146] performed SEM evaluation to explore the microstructure of composites without FA and composites with FA. The SEM analysis of samples was carried out at the age of 28-days. A dense, compacted, and uniformly distributed matrix was observed for the sample without FA, while cracked, porous, and partially reacted FA grains were observed in the sample containing FA. These observations are consistent with the reduced mechanical properties of FA-based composites, as discussed earlier. Ahad et al. [147] also studied and compared the microstructure of a reference mix without FA and a mix containing 30% FA, as depicted in Figure 7. Crack was observed in the matrix of the reference mix (Figure 7a). This may be due to the high heat of cement hydration resulting in the micro-cracks in the matrix. Though voids in the matrix are less and a denser and compacted matrix can be observed. Micro-cracks are not observed in the matrix of composite containing FA because FA addition reduced the heat of hydration; however, more voids and partially reacted FA particles are observed resulting in less dense matrix (Figure 7b). This also supports the detrimental effects of FA on cementitious composites.

**Figure 8:**
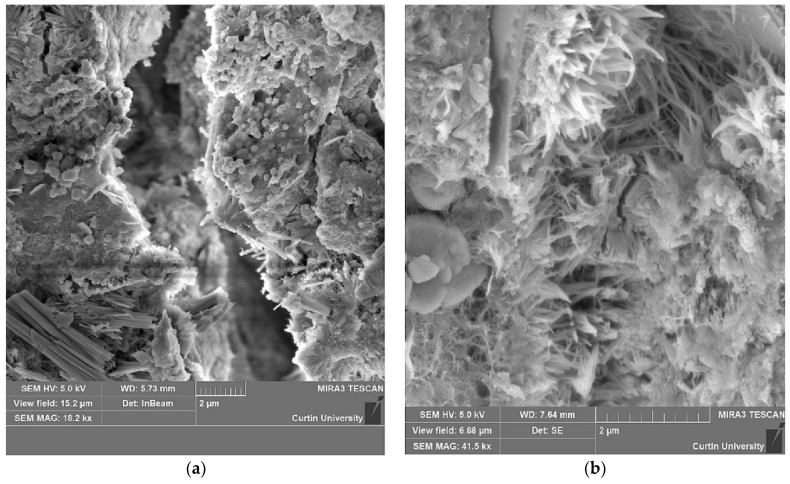
SEM micrographs of composites containing 40% fly ash as a biner at: (**a**) 28-days; (**b**) 180-days [122].

Saha [122] performed a microstructural study of FA concrete, and their micrographs are shown in Figure 8. Figure 8a illustrates the microstructural images of cracked cementitious material containing 40% FA as an SCM at the age of 28 days. Ettringite needles initiate to form in the voids of the binder matrix and on the surface of the FA. Smooth spherical FA grains are also visible, indicating that the FA has been hydrated during its first phase. FA has a somewhat spherical shape, and the existence of spherical grains in the microstructure of the matrix at the age of 28 days suggests that the FA grains have not reacted with the cement during the early hydration phase. Due to exposure to a harsh environment, the spherical form of FA rapidly spoils in cement mix and is substituted with ettringite needles [148,149]. This corroborates the idea that FA retards concrete hydration. Figure 8b illustrates the microstructure of FA concrete after 180 days of curing. The spherical grains were substituted by ettringite as a result of the pozzolanic reaction of FA. The voids between the aggregates are densely packed with ettringite needles. Additionally, the ettringite needles are longer, fill up the gaps in the cement mix; therefore, the ettringite needles fill the spaces between the aggregates by the pozzolanic reaction of FA. As a result, FA concrete produces a denser binder matrix than normal concrete [122].

## 4. Discussions

The challenges correlated with the manufacture and application of cement are well known. The growing need for concrete, and therefore cement, poses a severe danger to both the environment and human life. In this context, scholars are concentrating on the use of SCMs that can substitute cement in the manufacture of concrete, encouraging eco-friendly development. This study examined the usage of the most common industrial byproducts, i.e., FA, in cementitious composites as SCMs. This study highlighted and discussed the most critical sections, including the properties of FA, the characteristics of composites containing FA as SCMs, i.e., workability, compressive, split-tensile, and flexural strength, durability, and microstructural properties. Table 5 has been prepared to summarize the various parameters examined in this study for FA use as SCM. As can be noticed from the table, FA utilization contributes to construction sustainability. In addition to the sustainable benefits, FA has the further advantage of low heat of hydration. FA is a pozzolanic material and is utilized as SCM in cement-based composites. The use of FA as cement replacement in lower replacement ratios (up to 20%) has a positive effect on the mechanical and durability properties of composites, while at higher replacement ratios, it has a negative effect; moreover, the size of FA alters the properties of composites. The smaller size FA is preferable, as it has a more positive impact on the performance of composites.

FA consumption has increased in the concrete industry because of its benefits, which include reduced hydration heat and increased durability; however, due to the slow pozzolanic action, its contribution to strength begins only at a later age. Attempts have been undertaken to address this well-documented FA deficit using a variety of approaches. Chemical activation is one of these ways and can be accomplished using either alkali or sulphate. In alkali activation, the glass phases of FA are broken down to expedite the reaction at an early stage [150], whereas, in sulphate activation, sulphate combines with the aluminum oxide in the glass phase of FA to form ettringite [151]. In each of these instances, strength is developed at a young age [152]. Alkali activation of FA is a physicochemical method that converts powdered ash to a material with excellent cementitious characteristics [153,154], developing high mechanical strength and exceptional bonding to reinforcing bars [155]. The use of nanoparticles in FA-based cementitious composites to accelerate its early strength gain is becoming more common as a result of its benefits. The nanoparticles serve as nuclei for the cement, accelerating hydration and densifying the microstructure and interfacial transition zone, hence decreasing permeability [156]. Additionally, the combination of FA and nanomaterials enables the hydration product to be tightly bound, which is a critical element in accelerating the pozzolanic process since it compensates for the poor initial strength growth [157,158,159]; hence, the strength of the FA-based cementitious composites can be increased by various methods, including those covered above. For successful strength enhancement via alkali activation or nanoparticle addition, knowledge of the properties of FA is required. Ca/Si and Ca/Al ratios are regarded as critical factors in the formation of CSH gel in the case of nano addition and alumino silicate gel in the case of alkali activation, respectively. Using either of these approaches, it is possible to replace up to 60% of cement with FA without sacrificing strength or durability [94]. For high-volume FA concrete, a ternary blend of cement, FA, and nanomaterials can be advised.

## 5. Conclusions

The present study aimed to review the different aspects of the fly ash (FA) application as supplementary cementitious material (SCM) in cement-based materials. The influence of the FA characteristics of the mechanical, durability, and microstructural properties of the material is discussed. The various limitations of the FA use in higher proportions, and their potential solutions are described. This study reached the following conclusions:

- The influence of FA incorporation on the workability of fresh concrete was found to be inconsistent. Some studies reported an increase in the workability because of FA spherical shape, increased volume of the mix due to lower density of FA, and slower development of hydration products due to FA addition; however, some studies found a reduction in the workability of the fresh mix due to the smaller size and larger surface area of FA.
- Numerous studies have demonstrated that the application of FA inhibits composites’ early-age strength development; however, FA mostly improves the strength of concrete over time by consuming the Ca(OH)_2_ produced during cement hydration and producing secondary hydrates such as CSH; moreover, the mechanical strength of the composites improves when FA is incorporated in lower concentrations (up to 20%). In addition, finer particle size FA enhances while coarser particle size FA reduces the mechanical strength of composites.
- The resistance of composites to chloride penetration increases with the addition of FA, especially at later ages. The finer particle size FA decreases the interconnecting voids in the matrix, and pozzolanic action further improves the microstructure, resulting in increased resistance to chloride penetration.
- There is a contradiction regarding the influence of FA on the shrinkage of cementitious composites. FA incorporation may increase shrinkage due to the creation of a higher amount of small capillary spaces, which facilitates the evaporation of water, causing higher shrinkage. In contrast, due to finer particle size FA addition, the density and compactness of the mix increases, which prevents the internal moisture evaporation, causing reduced shrinkage.
- The influence of FA on sulphate resistance of composite is determined by the FA type. Generally, low calcium FA (Class C) exhibits more resistance to sulphate resistance than high calcium FA (Class F). This is because low calcium FA consumes more Ca(OH)_2_ to form CSH, compared to the high calcium FA.
- The incorporation of FA as SCM up to 20% content is beneficial to composites in terms of reducing water absorption; however, at higher replacement levels, the water absorption capacity increases.
- The slow early strength development is one of the major drawbacks of FA use in cementitious composites; however, this can be overcome by chemical activation (alkali/sulphate) and/or adding nanomaterials, and this can facilitate high volume usage of FA.
- Using FA as SCM will promote sustainable development due to reduction in CO_2_ emissions, preservation of natural resources, effective waste management, reduction in environmental pollution, and low heat of hydration.

## 6. Future Recommendations

After reviewing the different aspects of the FA application as SCM, this study suggests the following future research directions:

- Despite the numerous stated procedures for using FA in large quantities, 100% utilization of FA has not been accomplished due to the existence of some grey areas highlighted in this study that must be addressed in the future to increase FA’s usage as SCM. Future studies should be directed on resolving the stated disparities in shrinkage, water demand, and faster curing.
- While some attributes of structural performance, such as flexural and shear resistance, have been covered previously, additional research is necessary to support the case for FA concrete usage in reinforced concrete structures. These may include the bond strength of steel rebars, the examination of beam-column junctions, and seismic design.
- Since FA reactivity is based on multiple factors, trying to control one property may deteriorate other properties. For example, while decreasing the *w*/*b* in high volume FA concrete increases early/late age strength, the mix results in early-age cracking. Thus, it is critical to consider the combined effect of multiple parameters in order to maximize the benefits of FA and cement in an optimum planned mix.
- Presently, there is a growing tendency toward the production of geopolymers that include 100% FA [160]; however, to promote the applicability of geopolymer concrete, certain shortcomings such as curing regime, availability of activators, efflorescence, and alkali-silica reaction [39] must be addressed. In this respect, it is reported that designed FA concrete, which can replace up to 60% of cement, is a superior alternative in terms of strength and durability.
- Based on the comprehensive examination of FA as SCM, it is proposed that further fly ash classifications be added to the existing ASTM classifications.

## Figures and Tables

**Table 1:** Element oxides range in fly ash types.

Element Oxides	Range of Element Oxides (%)	
	Class F	Class C
SiO_2_	37.0–62.1	11.8–46.4
Al_2_O_3_	16.6–35.6	2.6–20.5
Fe_2_O_3_	2.6–21.2	1.4–15.6
CaO	0.5–14.0	15.1–54.8
MgO	0.3–5.2	0.1–6.7
SO_3_	0.02–4.7	1.4–12.9
Na_2_O	0.1–3.6	0.2–2.8
K_2_O	0.1–4.1	0.3–9.3
TiO_2_	0.5–2.6	0.6–1.0
P_2_O_5_	0.1–1.7	0.2–0.4
MnO	0.03–0.1	0.3–0.2
LOI	0.3–32.8	0.3–11.7

**Table 3:** Split-tensile strength (28-days) of composites containing fly ash.

Fly Ash		
Reference	Replacement Ratio (%)	Split-Tensile Strength (MPa)
Barbuta et al. [107], without fibers	0	1.72
	10	1.58
	15	1.58
	20	0.89
	30	1.21
	40	0.89
Barbuta et al. [107], with 0.25% and 30 mm long fibers	0	1.72
	10	1.51
	15	1.37
	20	1.05
	30	1.71
	40	1.02
Barbuta et al. [107], with 0.25% and 50 mm long fibers	0	1.72
	10	1.94
	15	1.45
	20	0.87
	30	1.82
	40	0.85
Gencel et al. [108], with 0% FSA	0	5.20
	10	4.70
	20	4.12
	30	3.63
Gencel et al. [108], with 25% FSA	0	5.31
	10	4.74
	20	4.17
	30	3.66
Gencel et al. [108], with 50% FSA	0	5.24
	10	4.78
	20	4.19
	30	3.78
Gencel et al. [108], with 75% FSA	0	5.30
	10	4.83
	20	4.22
	30	3.70

FSA: ferrochromium slag aggregate.

**Table 4:** Flexural strength (28-days) of composites containing fly ash.

Fly Ash		
Reference	Replacement Ratio (%)	Flexural Strength (MPa)
Barbuta et al. [107], without fibers	0	1.82
	10	2.19
	15	1.62
	20	1.53
	30	1.62
	40	1.04
Barbuta et al. [107], with 0.25% and 30 mm long fibers	0	1.82
	10	2.01
	15	1.82
	20	2.16
	30	1.97
	40	1.67
Barbuta et al. [107], with 0.25% and 50 mm long fibers	0	1.82
	10	2.01
	15	2.08
	20	2.49
	30	2.05
	40	1.96
Paliwal and Maru [115]	0	3.48
	5	3.88
	10	4.44
	15	4.14
	20	3.62
Huang et al. [109], 35 MPa concrete and low LOI fly ash	0	5.1
	20	5.3
	40	5.2
	60	5
	80	3.7
Huang et al. [109], 35 MPa concrete and high LOI fly ash	0	5.1
	20	5
	40	5.1
	60	4.5
	80	3.2

LOI: loss on ignition.

**Table 5:** Comparison of various aspects of utilizing silica fume and fly ash in cementitious materials.

Aspect	Detail
Sustainability	Reduction in CO_2_ emission Effective waste management Decreases environmental pollution Preserve natural resources Cost-effective Low heat of hydration
Influence on material properties	Inconsistent influence on workability Enhances the performance of composites when used in lower replacement ratios (up to 20%) Negative influence on material’s performance at higher proportions
Limitations	Utilization at higher replacement levels is not preferable Low early strength development

## Data Availability

Not applicable.
